# The crystal structure of the Dess–Martin periodinane

**DOI:** 10.3762/bjoc.8.172

**Published:** 2012-09-12

**Authors:** Albert Schröckeneder, Desiree Stichnoth, Peter Mayer, Dirk Trauner

**Affiliations:** 1 Department of Chemistry, Ludwig-Maximilians-Universität München, Butenandtstraße 5-13, 81377 München, Germany. Tel: +49 (0)89 2180 77800; Fax: +49 (0)89 2180 77972

**Keywords:** crystal structure, Dess–Martin periodinane, halogen bonds, hypervalent iodine, oxidant

## Abstract

We report the elusive X-ray structure of the Dess–Martin periodinane (DMP), a hypervalent iodine reagent popular amongst synthetic chemists. In the solid state, the highly crystalline compound forms an intricate coordination polymer held together by intermolecular halogen and hydrogen bonds.

## Introduction

The so-called Dess–Martin periodinane (DMP, 1,1,1-triacetoxy-1,1-dihydro-1,2-benziodoxol-3(1*H*)-one, **1**) has emerged as one of the most useful reagents for the oxidation of primary and secondary alcohols to the corresponding aldehydes and ketones [1–4]. Its solubility in organic solvents, high reactivity under mild conditions, and lack of toxic or unpleasant byproducts renders it often preferable over other oxidation reagents and has ensured its continued popularity among synthetic chemists.

**Figure 1:**
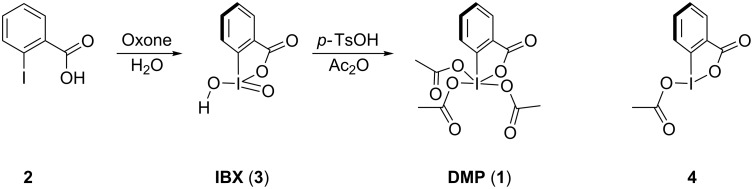
The chemical structures of DMP (**1**), 2-iodobenzoic acid (**2**), IBX (**3**) and **4**.

As depicted in Figure 1, DMP (**1**) is usually prepared from 2-iodobenzoic acid (**2**) in a two-step procedure, which involves oxidation with Oxone (a formulation of peroxomonosulfate) [5], followed by heating of the intermediary iodine(V) species IBX (**3**) with acetic anhydride and catalytic amounts of *p*-toluenesulfonic acid [6]. Upon repeating this procedure with an older and less-active batch of Oxone, we obtained well-defined single crystals of acetoxy-1,2-benziodoxolin-3-one (**4**) as a byproduct. We quickly established that the crystal structure of this partially oxidized compound had already been described by Gougoutas and Clardy four decades ago [7] and that the X-ray structure of IBX (**3**) was also known [8].

Browsing the Cambridge Crystallographic Data Centre, however, we were surprised to find that no X-ray structure of **1** itself has been published to date. We thus set out to investigate a range of crystallization conditions to obtain the first X-ray crystal structure of DMP (**1**). This could serve as a basis for computational investigations concerning the mechanism of the Dess–Martin oxidation.

## Results and Discussion

The difficulty in obtaining suitable single crystals for X-ray diffraction analysis of DMP (**1**) may lie in the high crystallinity of the compound. Indeed, microcrystalline **1** precipitates from the acetic anhydride solution upon cooling at the end of the procedure used for its preparation. Various recrystallization methods also yielded **1** as a microcrystalline powder that was unsuitable for single-crystal X-ray analysis. However, the standard protocol for the preparation of **1** requires filtration and washing of the filter cake with ether. The combined filtrates thus consist of a solution of residual **1** and various byproducts, including iodine(III) intermediates, in a mixed solution of ether and acetic anhydride. We reasoned that the presence of these impurities may slow down nucleation and allow for the growth of larger crystals. Indeed, slow evaporation of the filtrate under a constant stream of nitrogen at ambient temperature over the course of four days did yield single crystals suitable for X-ray analysis.

**Figure 2:**
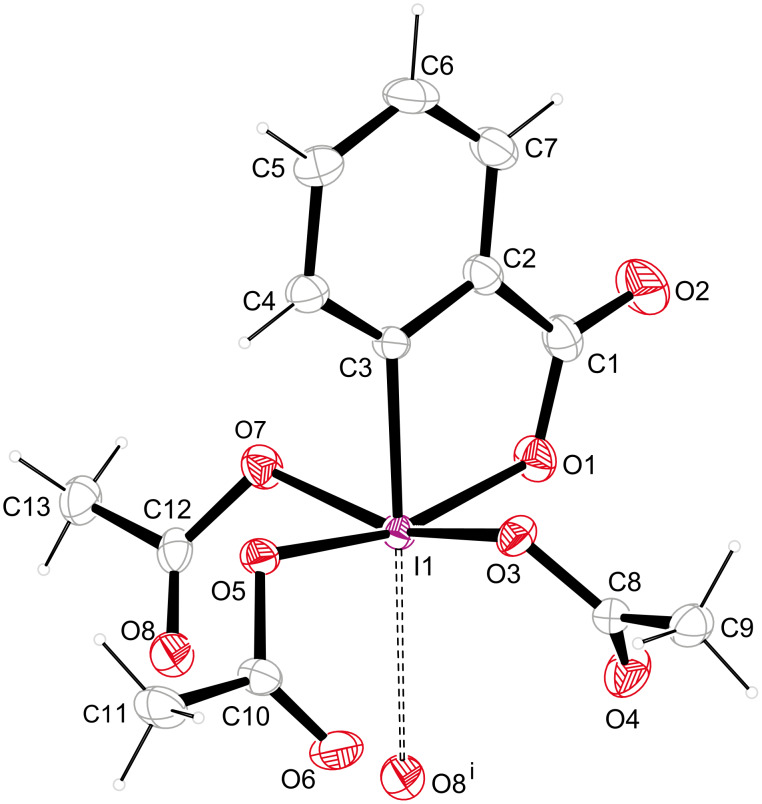
ORTEP diagram (50% probability level) of **1** with numbering scheme.

DMP (**1**) crystallizes in a triclinic unit cell, which is occupied by two molecules. An ORTEP plot and numbering scheme for monomeric DMP (**1**) with 50% probability ellipsoids is shown in Figure 2. Important bond lengths and angles are provided in Table 1.

**Table 1:** Selected bond lengths and angles in **1**.

iodine bond lengths (Å)		iodine angles (°)	

I1–C3	2.1025(16)	O1–I1–O3	92.84(5)
I1–O1	2.0888(14)	O1–I1–O5	165.06(5)
I1–O3	2.0656(13)	O1–I1–O7	88.66(5)
I1–O5	2.0670(13)	O1–I1–C3	79.66(6)
I1–O7	2.1141(13)	O3–I1–O5	84.32(5)
		O3–I1–O7	159.77(5)
		O3–I1–C3	80.50(6)
		O5–I1–O7	89.05(5)
		O5–I1–C3	85.40(6)
		O7–I1–C3	79.93(6)

Several structural features of monomeric **1** are noteworthy. The central iodine atom resides in a distorted octahedral environment that is in accordance with a simple VSEPR model. The equatorial positions are occupied by acetoxy groups, whereas the apical positions are occupied by the phenyl ring and the lone electron pair. Due to the steric demands of the electron pair, which is engaged in supramolecular interactions (see below), the acetoxy substituents are pushed toward the phenyl ring. As a consequence, the iodine atom lies 0.315(1) Å below a plane formed by oxygens O1, O3, O5 and O7 and all iodine–oxygen bonds form an acute angle with the iodine–carbon bond in the apical position. (O1–I1–C3 = 79.66(6)°, O3–I1–C3 = 80.50(6)°, O5–I1–C3 = 85.40(6)°, O7–I1–C3 = 79.93(6)°). All three acetoxy groups are bound in a covalent η-1 fashion, showing typical iodine–oxygen bond lengths (I1–O3 = 2.0656(13) Å, I1–O5 = 2.0670(13) Å, I1–O7 = 2.1141(13) Å) [9]. The length of the iodine–oxygen bond of the iodoxolone ring lies also well within the range of a covalent iodine–oxygen bond (I1–O1 = 2.0888(14) Å). The iodine–carbon bond has a length of I1–C3 = 2.1025(16) Å and forms an angle of 79.66(6)° with the I1–O1 bond. Interestingly, the I1–O1 bond (2.0888(14) Å) in **1** is shorter than the I1–C3 bond (2.1025(16) Å). By contrast, the respective bond lengths in iodoxolone **4** are 2.3236 Å and 2.1061 Å [7], and in iodoxolone **3** they are 2.1289 Å and 2.0121 Å [8].

It is worth noting that the two lateral acetoxy groups in **1** are bound to the central iodine atom with significantly different bond lengths (I1–O5 = 2.0670(13) Å, I1–O7 = 2.1141(13) Å), which desymmetrizes the molecule overall. This desymmetrization cannot be attributed to crystal-packing effects since it is also apparent in a structural model of **1** calculated by using DFT theory at the PBE0/LANL2DZdp level [10]. Overall this structure, calculated in the gas phase and at room temperature, is in good agreement with our experimentally determined X-ray structure (determined at *T* = 173 K), as the geometry around the central iodine atom is very similar. A closer look at the iodoxolone ring in the X-ray structure of **1**, however, reveals that the substituents O1 and O5 are not located in the least-square plane of the phenyl ring. The O1– and O5–iodine bonds deviate from the least-square plane by 5.34(8)° and 5.26(8)° respectively. This distortion is not evident in the structure calculated by Mocci and coworkers [10].

**Figure 3:**
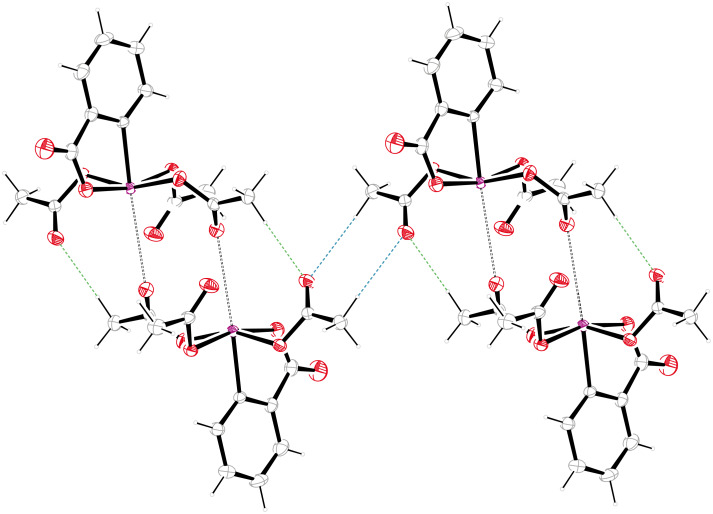
Supramolecular structure of **1** with halogen bonds and selected hydrogen bonds.

**Figure 4:**
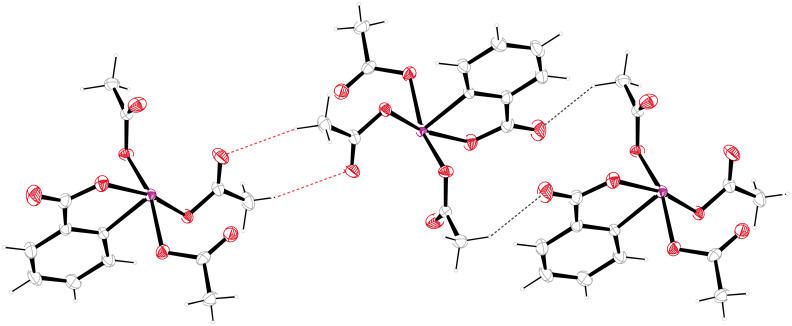
Further hydrogen-bond interactions in the supramolecular structure of **1**.

**Figure 5:**
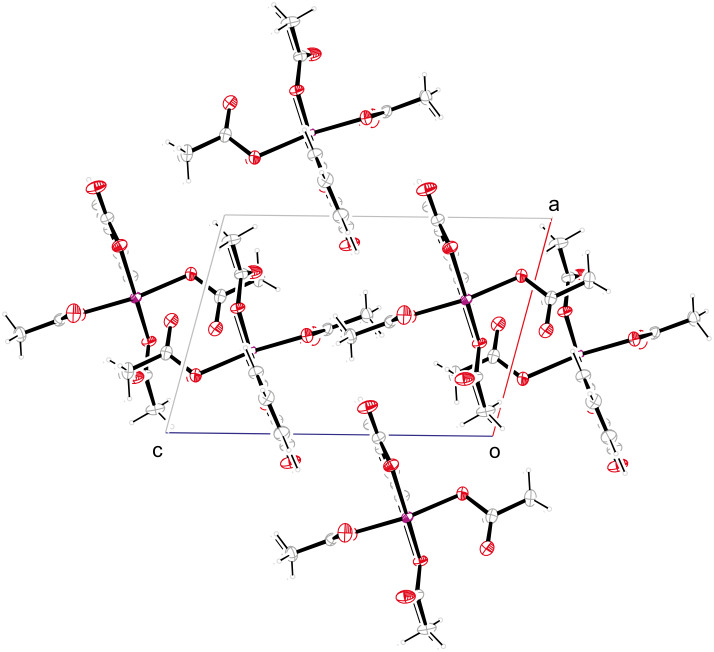
Supramolecular structure of **1** viewed along axis *b*.

The supramolecular structure of **1**, which has not been calculated before, is depicted in Figure 3, Figure 4 and Figure 5. The unit cell is occupied by a centrosymmetric dimer that is held together, in part, by two intermolecular halogen–oxygen bonds between the iodine and a carbonyl group of the adjacent molecule. The intermolecular iodine–oxygen distance of 3.3 Å lies well below the sum of the van der Waals radii (3.46 Å) [11]. Angles of 159.1° (C3a–I1a–O8b) and 120.2° (I1a-O8b-C12b) are also in accordance with the presence of halogen bonds [12]. Similar bonds between iodine atoms and sp^2^-hybridized oxygen atoms have been observed in a range of other hypervalent iodine species [7–8,13–17], as well as iodine(I) compounds [18–19]. The ability to form halogen bonds may also account for the high solubility of DMP (**1**) in organic solvents.

In addition to halogen bonds, the dimer is stabilized by two weak hydrogen bonds of the type C13-H13A^…^O4 (green dashed bonds in Figure 3). In terms of graph-set analysis [20], the resulting 16-membered ring may be assigned a R_2_^2^(16) descriptor. The dimers themselves are linked by two weak hydrogen bonds of the type C9–H9A^…^O4 (blue dashed bonds in Figure 3, Table 2). The descriptor for this linking cyclic hydrogen bond is R_2_^2^(8). As a consequence of the two different types of hydrogen bonds, the dimers form infinite chains along the *c*-axis. The chains appear on the binary level with a C_2_^1^(10) descriptor, as the shortest repeating unit of the chain consists of ten atoms including two donor atoms and only one acceptor atom. There are two additional weak hydrogen bonds, which link adjacent molecules by cyclic centrosymmetric hydrogen-bond systems (Figure 4).

**Table 2:** Halogen and hydrogen bonds in **1**.

bond lengths in the crystal lattice of **1** (Å)	

I1^…^O8	3.2635(15)
C9-H9A^…^O5	2.877(2)
C9-H9C^…^O2	3.312(3)
C11-H11A^…^O6	3.470(3)
C13-H13A^…^O4	3.265(3)

A 16-membered ring with a R_2_^2^(16) descriptor is established by involvement of the iodoxolone carbonyl in a weak hydrogen bond C9–H9C^…^O2 (black dashed lines in Figure 4). An eight-membered ring comparable to the one discussed above is formed by two hydrogen bonds of the type C11–H11A^…^O6 (red dashed lines in Figure 3) resulting in a R_2_^2^(8) descriptor. The combination of the two different hydrogen bonds depicted in Figure 3 and Figure 4 lead to the formation of infinite chains along [111], which appear on the binary level with a C_2_^2^(16) descriptor. In the packing, the phenyl planes are arranged approximately parallel to the *b*-axis with a deviation angle of only 1.58(7)° (Figure 5). There are, however, no π-stacking interactions. Least-square planes of the four oxygen atoms bound covalently to iodine are nearly parallel to the *c*-axis (deviation angle 0.79(4)°).

## Conclusion

In conclusion, we have determined the solid-state structure of the Dess–Martin periodinane (**1**), a popular reagent in organic synthesis. The similarity of our experimentally determined structure with a calculated gas-phase structure of monomeric **1** underscores the power of density functional theory. A network of halogen and hydrogen bonds in the supramolecular structure explains the high crystallinity of **1** and has implications with respect to the mechanism of the Dess–Martin oxidation. Our X-ray structure will serve as a starting point for detailed quantum mechanical calculations to address this topic.

## Experimental

Crystallographic data for **1**: Data collected by means of an Oxford Diffraction Xcalibur 3 diffractometer, Mo Kα radiation (λ = 0.71073 Å), *T* = 173 K; C_13_H_13_IO_8_, *M*_r_ = 424.142 g/mol, colorless block, 0.30 × 0.21 × 0.15 mm^3^, triclinic, *P*−1, *a* = 8.3829(4), *b* = 8.4906(6), *c* = 11.6195(8) Å, α = 100.659(6)°, β = 99.289(5)°, γ = 111.040(5)°, *V* = 734.80(8) Å^3^, *Z* = 2, ρ = 1.917 g/cm^3^, μ = 2.218 mm^−1^; 6431 reflections, 4411 independent reflections (4154 with *I* ≥ 2σ(*I*)), *R*_int_ = 0.0141, 202 parameters, final *R*_1_ = 0.0187 for data with *I* ≥ 2σ(*I*), *R*_w_(*F*^2^) = 0.0479; the hydrogen atoms were positioned geometrically (C–H = 0.98 Å for methyl, 0.95 Å for phenyl H) and treated as riding on their parent atoms; the methyl groups were allowed to rotate along the acetate C–C bonds to best fit the experimental electron density. CCDC 866010.

**1-Hydroxy-1,2-benziodoxol-3(1*H*)-one (IBX) (3):** 2-iodobenzoic acid (100 g, 403 mmol) was added to a solution of Oxone (2KHSO_5_/KHSO_4_/K_2_SO_4_) (322 g, 524 mmol) in water (2 L) and stirred at 80 °C for 4 h. The suspension was cooled to 4 °C under slow stirring. The mixture was filtered and the white precipitate was washed with water (2 × 100 mL) and acetone (2 × 100 mL) then dried under high vacuum to yield a colorless powder of IBX (**3**) (98 g, 87%). This material was used in the next step without further purification.

**1,1,1-Triacetoxy-1,1-dihydro-1,2-benziodoxol-3(1*H*)-one (DMP) (1):** IBX (**3**) (98 g, 350 mmol) was subsequently added to acetic anhydride (400 mL) and *p*-TsOH·H_2_O (400 mg, 2.10 mmol) and stirred at 80 °C. After 2 h the clear solution was cooled to 4 °C and the white precipitate was filtered off, washed with ether (2 × 100 mL) and dried under high vacuum to yield 126 g (85%) of DMP (**1**). To obtain suitable crystals for X-ray analysis, all filtrates were combined in a filter flask. A D3 glass frit was fitted, and ether was allowed to evaporate under a gentle stream of nitrogen at ambient temperature over the course of four days. Obtained single crystals of **1** were washed with anhydrous ether at 0 °C and stored under argon. ^1^H NMR (300 MHz, CDCl_3_) δ 8.33–8.24 (m, 2H), 8.11–8.04 (m, 1H), 7.90 (td, *J* = 0.9, 7.4 Hz, 1H), 2.32 (s, 3H), 1.99 (s, 6H); ^13^C NMR (75 MHz, CDCl_3_) δ 175.82, 174.12, 166.23, 142.32, 135.87, 133.94, 131.87, 126.59, 126.02, 20.54, 20.39; IR (ATR) 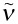: 1699.9, 1670.6.

## Supporting Information

File 1 Detailed crystallographic data of Dess–Martin periodinane (**1**).
