# Supplementary material for: The crystal structure of the Dess–Martin periodinane
Source: Beilstein J Org Chem. 2012 Sep 12;8:1523–7. doi: 10.3762/bjoc.8.172 (PMC3458777; doi:10.3762/bjoc.8.172)
Supplement: File 1 — Detailed crystallographic data of Dess–Martin periodinane (1). [file Beilstein_J_Org_Chem-08-1523-s001.pdf]

Supporting Information

for

The crystal structure of the Dess–Martin periodinane

Albert Schröckeneder, Desiree Stichnoth, Peter Mayer and Dirk Trauner *

Address: Department of Chemistry, Ludwig-Maximilians-Universität München, Butenandtstraße 5-13, 81377 München, Germany. Tel: +49 (0)89 2180 77800; Fax: +49 (0)89 2180 77972

Email: Dirk Trauner* - dirk.trauner@lmu.de

*Corresponding author

Detailed crystallographic data of Dess–Martin periodinane (1)

Contents

Experimental procedure for **1**	S3
^{1}H NMR spectrum of **1**	S4
^{13}C NMR spectrum of **1**	S5
Crystallographic data of **1**	S6
ORTEP drawing of **1**	S18

Experimental procedure for the synthesis of the Dess-Martin-Periodinane (**1**)

Proton (^{1}H) and carbon (^{13}C) spectra were recorded at 20 °C on Varian Mercury spectrometers operating at 300 Hz for proton nuclei (75 MHz, for carbon nuclei). For ^{1}H NMR spectra, multiplicity is defined as: s = singlet; d = doublet; t = triplet; m = multiplet or combinations of the above. The residual $CHCl_3$ peak (δ = 7.26 ppm) was used as references for the ^{1}H NMR spectrum. The central peak (δ = 77.13 ppm) of the $CDCl_3$ 'triplet' was used as references for the proton-decoupled ^{13}C NMR spectrum.

The infrared spectrum was recorded on a Perkin-Elmer BXII-FTIR spectrometer. The sample was analyzed as neat material.

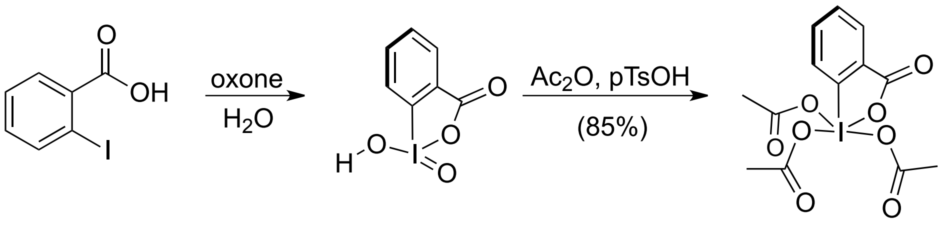

2 **3** **1**

2-iodobenzoic acid (100 g, 403 mmol) was added to a solution of oxone® ($2\cdot KHSO_5$-$KHSO_4$-K_2SO_4) (322 g, 524 mmol) in water (2 L) and stirred at 80 °C for 4 h. The suspension was cooled to 4 °C under slow stirring. The mixture was filtered and the white precipitate was washed with water (2x 100 ml) and acetone (2x 100 ml) and then dried under high vacuum to yield a colorless powder of IBX (**3**) (98 g, 87%). IBX (**3**) (98 g, 350 mmol) was subsequently added to acetic anhydride (400 mL) and *p*TsOH · H_2O (400 mg, 2.10 mmol) and stirred at 80 °C. After 2 h the clear solution was cooled to 4 °C and the white precipitate was filtered off, washed with ether (2x 100 ml) and dried under high vacuum to yield 126 g (85%) of DMP (**1**).

To obtain suitable crystals for X-ray analysis all filtrates were combined in a filter flask. A D3 glass frit was fitted and ether was allowed to evaporate under a gentle stream of nitrogen at ambient temperature over the course of four days. Obtained single crystals of **1** were washed with anhydrous ether at 0 °C and stored under Argon.

^{1}H NMR (300 MHz, $CDCl_3$) δ = 8.33 – 8.24 (m, 2H), 8.11 – 8.04 (m, 1H), 7.90 (td, J = 0.9, 7.4 Hz, 1H), 2.32 (s, 3H), 1.99 (s, 6H).

^{13}C NMR (75 MHz, $CDCl_3$) δ = 175.82, 174.12, 166.23, 142.32, 135.87, 133.94, 131.87, 126.59, 126.02, 20.54, 20.39.

IR (ATR): $\tilde{\nu}$ = 1699.9, 1670.6.

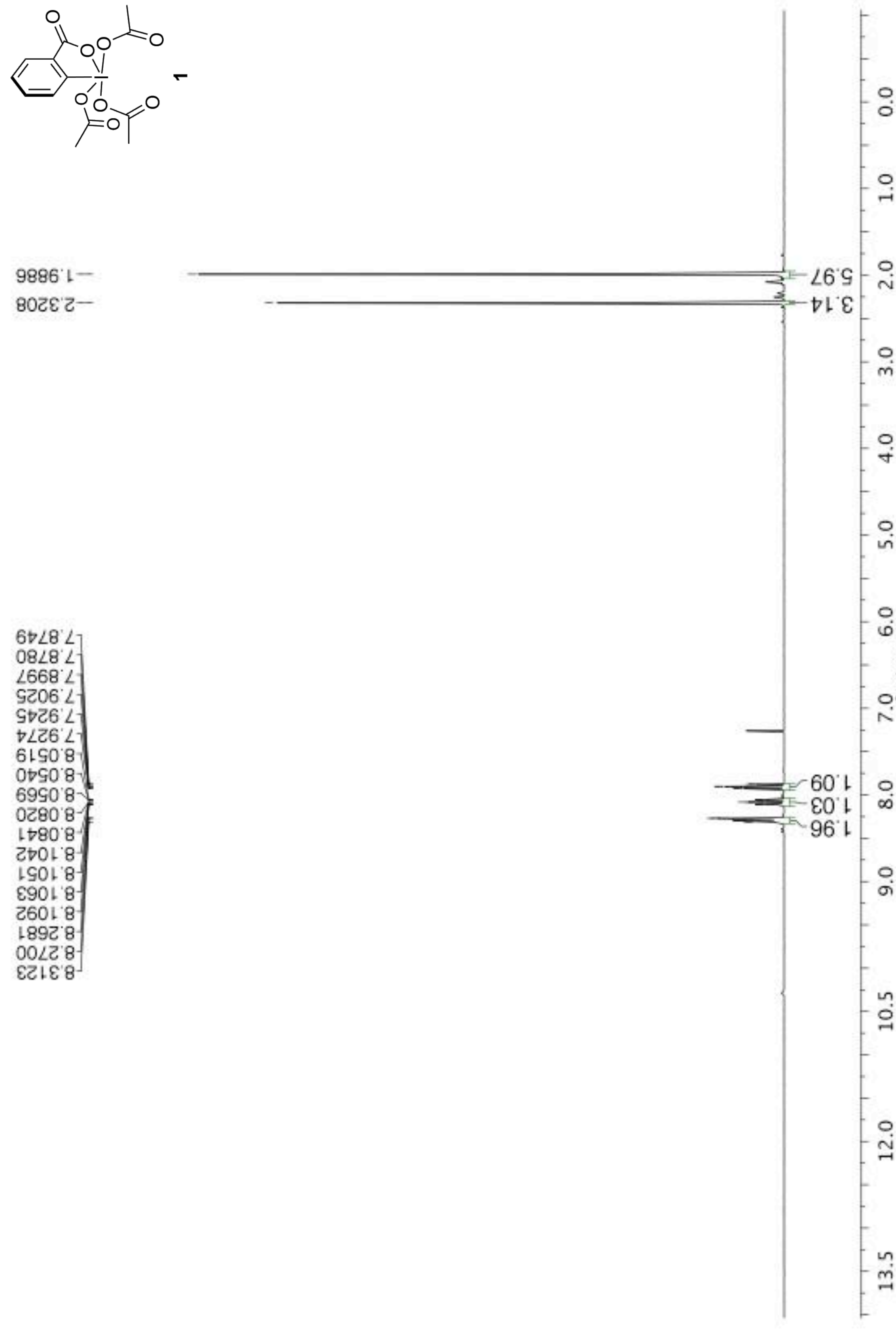
1
1.9886
2.3208
7.8749
7.8780
7.8997
7.9025
7.9245
7.9274
8.0519
8.0540
8.0569
8.0820
8.0841
8.1042
8.1051
8.1063
8.1092
8.2681
8.2700
8.3123
5.97
3.14
1.09
1.03
1.96
f1 (ppm)

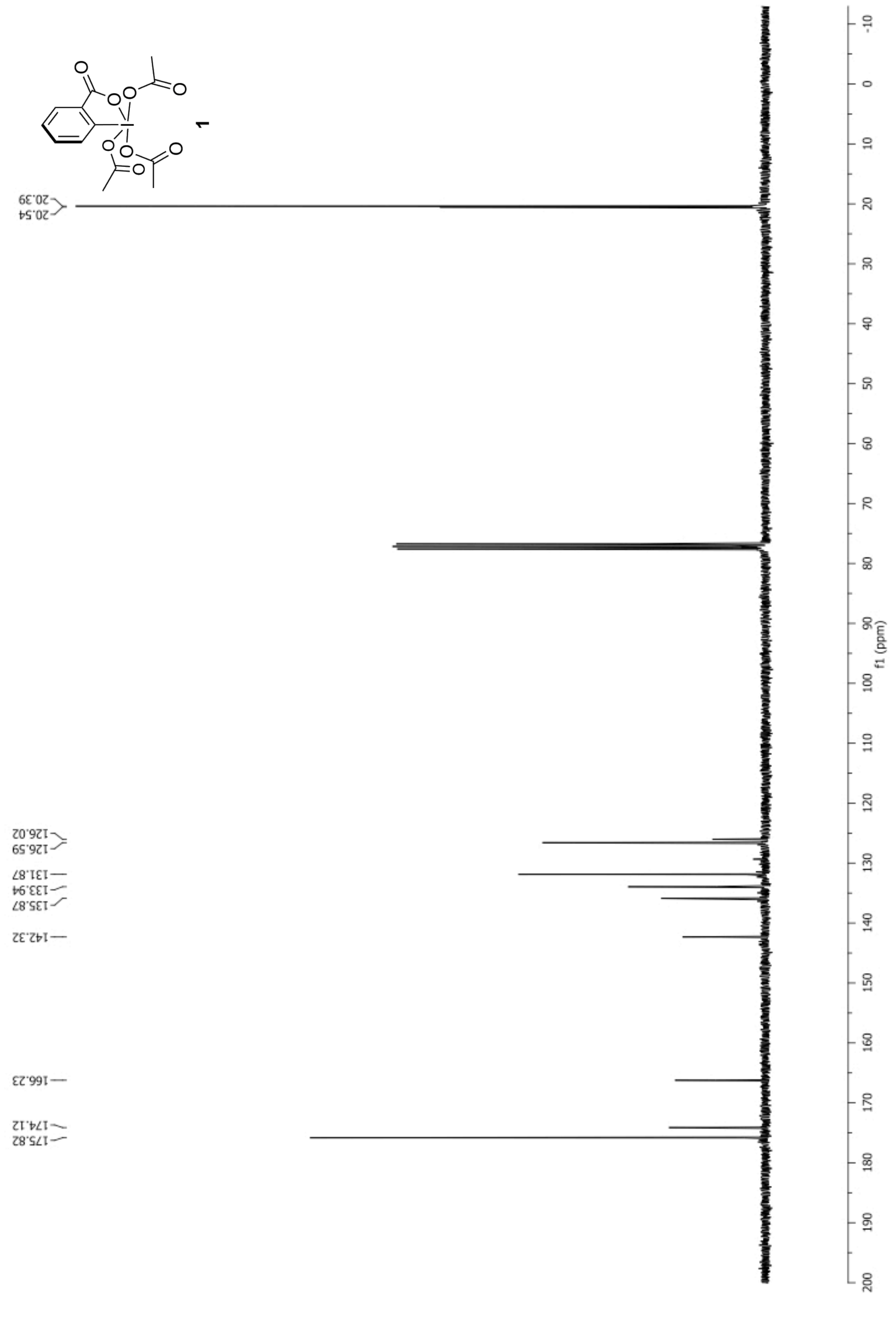
1
20.39
20.54
126.02
126.59
131.87
133.94
135.87
142.32
166.23
174.12
175.82
f1 (ppm)

Crystallographic Data

Table S1. Crystal Data and Details of the Structure Determination

for: qo007 P -1 R = 0.02

	**1**
net formula	$C_{13}H_{13}IO_8$
M_r/g mol^{-1}	424.142
crystal size/mm	0.30 × 0.21 × 0.15
T/K	173(2)
radiation	MoKα
diffractometer	'Oxford XCalibur'
crystal system	triclinic
space group	*P*1bar
a/Å	8.3829(4)
b/Å	8.4906(6)
c/Å	11.6195(8)
α/°	100.659(6)
β/°	99.289(5)
γ/°	111.040(5)
V/Å^3	734.80(8)
Z	2
calc. density/g cm^{-3}	1.9170(2)
μ/mm^{-1}	2.218
absorption correction	'multi-scan'
transmission factor range	0.611–0.717
refls. measured	6431
R_{int}	0.0141

mean $\sigma(I)/I$	0.0263
θ range	4.29–30.50
observed refls.	4154
x, y (weighting scheme)	0.0244, 0.0931
hydrogen refinement	constr
refls in refinement	4411
parameters	202
restraints	0
$R(F_{obs})$	0.0187
$R_w(F^2)$	0.0479
S	1.060
shift/error$_{max}$	0.001
max electron density/e Å^{-3}	0.548
min electron density/e Å^{-3}	−0.590

Table S2 - Final Coordinates and Equivalent Isotropic Displacement

Parameters of the non-Hydrogen atoms

for: qo007 P -1 R = 0.02

Atom	x	y	z	U(eq) [Ang^2]
I1	0.62320(1)	0.79523(1)	0.19489(1)	0.0152(1)
O1	0.86019(16)	0.79844(18)	0.28734(12)	0.0236(3)
O2	1.12754(18)	0.9733(2)	0.40553(15)	0.0384(5)
O3	0.54689(16)	0.85251(16)	0.35238(11)	0.0197(3)
O4	0.54732(19)	0.59724(18)	0.37384(13)	0.0295(4)
O5	0.42444(15)	0.86188(16)	0.11833(11)	0.0192(3)
O6	0.25756(17)	0.59897(18)	0.13599(14)	0.0306(4)
O7	0.73447(16)	0.83331(17)	0.04621(11)	0.0224(3)
O8	0.48396(17)	0.61850(18)	-0.07201(12)	0.0255(4)
C1	0.9868(2)	0.9559(3)	0.34624(16)	0.0234(5)
C2	0.9389(2)	1.1042(2)	0.32833(15)	0.0201(4)
C3	0.7759(2)	1.0662(2)	0.25412(14)	0.0165(4)
C4	0.7211(2)	1.1915(2)	0.22474(17)	0.0227(5)
C5	0.8378(3)	1.3662(2)	0.27538(18)	0.0273(5)
C6	1.0008(3)	1.4085(3)	0.35153(19)	0.0297(5)
C7	1.0531(2)	1.2793(3)	0.37890(18)	0.0276(5)
C8	0.5171(2)	0.7244(2)	0.40938(15)	0.0196(4)
C9	0.4489(3)	0.7614(3)	0.51805(17)	0.0257(5)
C10	0.2677(2)	0.7289(2)	0.10250(16)	0.0208(4)
C11	0.1131(2)	0.7594(3)	0.04381(19)	0.0296(5)

C12	0.6320(2)	0.7251(2)	-0.06104(15)	0.0200(4)
C13	0.7170(3)	0.7567(3)	-0.16363(16)	0.0270(5)

U(eq) = 1/3 of the trace of the orthogonalized U Tensor

Table S3 - Hydrogen Atom Positions and Isotropic Displacement Parameters

for: qo007 P -1 R = 0.02

Atom	x	y	z	U(iso) [Ang^2]
H4	0.60890	1.16070	0.17240	0.0270
H5	0.80510	1.45680	0.25740	0.0330
H6	1.07800	1.52800	0.38560	0.0360
H7	1.16510	1.30970	0.43130	0.0330
H9A	0.41500	0.65850	0.55030	0.0390
H9B	0.34580	0.78830	0.49520	0.0390
H9C	0.54120	0.86150	0.57990	0.0390
H11A	0.03310	0.65460	-0.02020	0.0440
H11B	0.15430	0.85880	0.00880	0.0440
H11C	0.05050	0.78480	0.10430	0.0440
H13A	0.66670	0.64930	-0.22980	0.0400
H13B	0.84440	0.79080	-0.13570	0.0400
H13C	0.69540	0.85050	-0.19230	0.0400

==

The Temperature Factor has the Form of Exp(-T) Where

T = 8*(Pi**2)*U*(Sin(Theta)/Lambda)**2 for Isotropic Atoms

Table S4 - (An)isotropic Displacement Parameters

for: qo007 P -1 R = 0.02

Atom	U(1,1) or U	U(2,2)	U(3,3)	U(2,3)	U(1,3)	U(1,2)
----	------	------	------	------	------	------
I1	0.0151(1)	0.0140(1)	0.0160(1)	0.0037(1)	0.0014(1)	0.0063(1)
O1	0.0209(6)	0.0244(6)	0.0261(6)	0.0069(5)	0.0002(5)	0.0122(5)
O2	0.0219(6)	0.0435(9)	0.0446(9)	0.0119(8)	-0.0055(6)	0.0131(6)
O3	0.0259(6)	0.0164(6)	0.0194(6)	0.0065(5)	0.0082(5)	0.0094(5)
O4	0.0452(8)	0.0219(7)	0.0270(7)	0.0089(5)	0.0106(6)	0.0178(6)
O5	0.0144(5)	0.0173(6)	0.0240(6)	0.0068(5)	0.0001(4)	0.0056(4)
O6	0.0214(6)	0.0214(7)	0.0452(8)	0.0143(6)	0.0025(6)	0.0040(5)
O7	0.0230(6)	0.0249(6)	0.0167(6)	0.0030(5)	0.0052(5)	0.0079(5)
O8	0.0274(6)	0.0235(7)	0.0235(6)	0.0056(5)	0.0033(5)	0.0095(5)
C1	0.0191(7)	0.0294(9)	0.0211(8)	0.0065(7)	0.0025(6)	0.0102(7)
C2	0.0163(7)	0.0232(8)	0.0185(7)	0.0046(6)	0.0035(6)	0.0061(6)
C3	0.0155(7)	0.0146(7)	0.0164(7)	0.0033(6)	0.0033(5)	0.0034(6)
C4	0.0209(8)	0.0184(8)	0.0265(9)	0.0066(7)	0.0021(6)	0.0066(6)
C5	0.0308(9)	0.0181(8)	0.0313(10)	0.0067(7)	0.0069(8)	0.0080(7)
C6	0.0277(9)	0.0178(8)	0.0317(10)	-0.0004(7)	0.0058(8)	-0.0003(7)
C7	0.0196(8)	0.0283(10)	0.0258(9)	0.0030(8)	0.0006(7)	0.0033(7)
C8	0.0204(7)	0.0167(7)	0.0177(7)	0.0049(6)	0.0021(6)	0.0039(6)
C9	0.0308(9)	0.0227(9)	0.0215(8)	0.0062(7)	0.0101(7)	0.0065(7)
C10	0.0168(7)	0.0183(8)	0.0237(8)	0.0033(6)	0.0022(6)	0.0054(6)
C11	0.0164(8)	0.0255(9)	0.0410(11)	0.0075(8)	-0.0020(7)	0.0064(7)
C12	0.0277(8)	0.0191(8)	0.0176(7)	0.0061(6)	0.0038(6)	0.0145(7)
C13	0.0374(10)	0.0253(9)	0.0187(8)	0.0060(7)	0.0080(7)	0.0125(8)

===

The Temperature Factor has the Form of Exp(-T) Where

T = 8*(Pi**2)*U*(Sin(Theta)/Lambda)**2 for Isotropic Atoms

T = 2*(Pi**2)*Sumij(h(i)*h(j)*U(i,j)*Astar(i)*Astar(j)), for Anisotropic Atoms. Astar(i) are Reciprocal Axial Lengths and h(i) are the Reflection Indices.

Table S5 - Bond Distances (Angstrom)

for: qo007 P -1 R = 0.02

I1	-O1	2.0888(14)	C5	-C6	1.385(3)
I1	-O3	2.0656(13)	C6	-C7	1.388(3)
I1	-O5	2.0670(13)	C8	-C9	1.495(3)
I1	-O7	2.1141(13)	C10	-C11	1.494(3)
I1	-C3	2.1025(16)	C12	-C13	1.502(3)
O1	-C1	1.338(3)	C4	-H4	0.9500
O2	-C1	1.210(3)	C5	-H5	0.9500
O3	-C8	1.345(2)	C6	-H6	0.9500
O4	-C8	1.211(2)	C7	-H7	0.9500
O5	-C10	1.347(2)	C9	-H9A	0.9800
O6	-C10	1.216(2)	C9	-H9B	0.9800
O7	-C12	1.341(2)	C9	-H9C	0.9800
O8	-C12	1.215(2)	C11	-H11A	0.9800
C1	-C2	1.491(3)	C11	-H11B	0.9800
C2	-C3	1.384(2)	C11	-H11C	0.9800
C2	-C7	1.395(3)	C13	-H13A	0.9800
C3	-C4	1.376(2)	C13	-H13B	0.9800
C4	-C5	1.397(2)	C13	-H13C	0.9800

Table S6 - Bond Angles (Degrees)

for: qo007 P -1 R = 0.02

O1	-I1	-O3	92.84(5)	O3	-C8	-C9	112.51(16)
O1	-I1	-O5	165.06(5)	O4	-C8	-C9	126.21(17)
O1	-I1	-O7	88.66(5)	O5	-C10	-O6	121.45(17)
O1	-I1	-C3	79.66(6)	O5	-C10	-C11	113.91(16)
O3	-I1	-O5	84.32(5)	O6	-C10	-C11	124.62(18)
O3	-I1	-O7	159.77(5)	O7	-C12	-O8	122.29(16)
O3	-I1	-C3	80.50(6)	O7	-C12	-C13	112.84(16)
O5	-I1	-O7	89.05(5)	O8	-C12	-C13	124.81(17)
O5	-I1	-C3	85.40(6)	C3	-C4	-H4	122.00
O7	-I1	-C3	79.93(6)	C5	-C4	-H4	121.00
I1	-O1	-C1	116.42(13)	C4	-C5	-H5	120.00
I1	-O3	-C8	113.04(11)	C6	-C5	-H5	120.00
I1	-O5	-C10	108.88(11)	C5	-C6	-H6	119.00
I1	-O7	-C12	114.84(11)	C7	-C6	-H6	119.00
O1	-C1	-O2	122.2(2)	C2	-C7	-H7	120.00
O1	-C1	-C2	113.64(16)	C6	-C7	-H7	120.00
O2	-C1	-C2	124.2(2)	C8	-C9	-H9A	109.00
C1	-C2	-C3	118.35(15)	C8	-C9	-H9B	109.00
C1	-C2	-C7	123.16(17)	C8	-C9	-H9C	109.00
C3	-C2	-C7	118.44(16)	H9A	-C9	-H9B	109.00
I1	-C3	-C2	111.58(12)	H9A	-C9	-H9C	109.00
I1	-C3	-C4	124.68(13)	H9B	-C9	-H9C	109.00
C2	-C3	-C4	123.73(16)	C10	-C11	-H11A	109.00
C3	-C4	-C5	117.04(18)	C10	-C11	-H11B	109.00

C4	-C5	-C6	120.59(19)	C10	-C11	-H11C	109.00
C5	-C6	-C7	121.1(2)	H11A	-C11	-H11B	109.00
C2	-C7	-C6	119.06(19)	H11A	-C11	-H11C	109.00
O3	-C8	-O4	121.27(16)	H11B	-C11	-H11C	109.00
C12	-C13	-H13A	109.0	H13A	-C13	-H13B	109.00
C12	-C13	-H13B	109.0	H13A	-C13	-H13C	109.00
C12	-C13	-H13C	109.0	H13B	-C13	-H13C	109.00

Table S7 - Torsion Angles (Degrees)

for: qo007 P -1 R = 0.02

O3	-I1	-O1	-C1	-74.77(13)
O7	-I1	-O1	-C1	85.04(13)
C3	-I1	-O1	-C1	5.03(13)
O1	-I1	-O3	-C8	-65.71(12)
O5	-I1	-O3	-C8	129.01(12)
O7	-I1	-O3	-C8	-159.57(14)
C3	-I1	-O3	-C8	-144.72(13)
O3	-I1	-O5	-C10	-76.68(11)
O7	-I1	-O5	-C10	122.46(11)
C3	-I1	-O5	-C10	-157.57(12)
O1	-I1	-O7	-C12	131.87(12)
O3	-I1	-O7	-C12	-133.53(16)
O5	-I1	-O7	-C12	-62.90(12)
C3	-I1	-O7	-C12	-148.40(13)
O1	-I1	-C3	-C2	-5.15(12)
O1	-I1	-C3	-C4	175.69(16)
O3	-I1	-C3	-C2	89.53(12)

O3	-I1	-C3	-C4	-89.62(15)
O5	-I1	-C3	-C2	174.52(13)
O5	-I1	-C3	-C4	-4.63(15)
O7	-I1	-C3	-C2	-95.63(13)
O7	-I1	-C3	-C4	85.22(15)
I1	-O1	-C1	-O2	177.92(15)
I1	-O1	-C1	-C2	-3.78(19)
I1	-O3	-C8	-O4	5.5(2)
I1	-O3	-C8	-C9	-175.66(13)
I1	-O5	-C10	-O6	3.1(2)
I1	-O5	-C10	-C11	-178.16(12)
I1	-O7	-C12	-O8	2.9(2)
I1	-O7	-C12	-C13	-179.75(13)
O1	-C1	-C2	-C3	-0.9(2)
O1	-C1	-C2	-C7	-178.31(17)
O2	-C1	-C2	-C3	177.34(18)
O2	-C1	-C2	-C7	0.0(3)
C1	-C2	-C3	-I1	5.0(2)
C1	-C2	-C3	-C4	-175.89(17)
C7	-C2	-C3	-I1	-177.53(14)
C7	-C2	-C3	-C4	1.6(3)
C1	-C2	-C7	-C6	176.30(18)
C3	-C2	-C7	-C6	-1.1(3)
I1	-C3	-C4	-C5	177.98(14)
C2	-C3	-C4	-C5	-1.1(3)
C3	-C4	-C5	-C6	0.0(3)
C4	-C5	-C6	-C7	0.5(3)
C5	-C6	-C7	-C2	0.1(3)

Table S8 - Contact Distances(Angstrom)

for: qo007 P -1 R = 0.02

I1	.O4	2.9166(15)	O1	.H11C_c	2.8700
I1	.O6	2.8060(16)	O2	.H9C_d	2.5800
I1	.O8	3.0140(14)	O2	.H7	2.7100
I1	.O8_a	3.2635(15)	O3	.H9C_e	2.8000
O1	.O4	3.013(2)	O4	.H7_d	2.7900
O2	.C1_d	3.204(2)	O4	.H13A_a	2.3300
O2	.C2_d	3.369(2)	O4	.H9A_f	2.5900
O2	.C8_d	3.238(2)	O5	.H4	2.3500
O2	.C9_d	3.312(3)	O6	.H11A_g	2.5500
O3	.O6	3.020(2)	O6	.H13A_a	2.7600
O3	.C9_e	3.330(3)	O7	.H11C_c	2.8200
O4	.O1	3.013(2)	O7	.H11B_h	2.6800
O4	.C13_a	3.265(3)	O8	.H4_h	2.6500
O4	.I1	2.9166(15)	O8	.H5_h	2.7600
O5	.O8	2.9693(19)	C1	.O2_d	3.204(2)
O6	.C8	3.288(2)	C1	.C1_d	3.466(3)
O6	.O3	3.020(2)	C2	.O2_d	3.369(2)
O6	.C12_a	3.223(2)	C4	.O8_h	3.282(2)
O6	.C13_a	3.173(3)	C4	.C9_e	3.531(3)
O6	.I1	2.8060(16)	C5	.O8_h	3.344(3)
O8	.O8_a	2.900(2)	C6	.C6_i	3.517(3)
O8	.C10	3.159(2)	C7	.C13_j	3.429(3)
O8	.I1_a	3.2635(15)	C8	.O2_d	3.238(2)
O8	.O5	2.9693(19)	C8	.O6	3.288(2)
O8	.C4_h	3.282(2)	C9	.C4_e	3.531(3)

O8	.C5_h	3.344(3)	C9	.O3_e	3.330(3)
O8	.I1	3.0140(14)	C9	.O2_d	3.312(3)
O1	.H5_b	2.7100	C10	.O8	3.159(2)
C12	.O6_a	3.223(2)	H6	.H9B_n	2.4500
C13	.C7_j	3.429(3)	H7	.O2	2.7100
C13	.O6_a	3.173(3)	H7	.O4_d	2.7900
C13	.O4_a	3.265(3)	H9A	.O4_f	2.5900
C4	.H11B_h	3.0700	H9B	.H6_k	2.4500
C7	.H13B_j	3.1000	H9C	.O2_d	2.5800
C7	.H13A_j	3.0900	H9C	.O3_e	2.8000
C9	.H6_k	2.9800	H11A	.O6_g	2.5500
C11	.H13B_l	2.9400	H11B	.O7_h	2.6800
C12	.H4_h	2.7800	H11B	.C4_h	3.0700
C13	.H4_h	3.0500	H11C	.O1_l	2.8700
H4	.O5	2.3500	H11C	.O7_l	2.8200
H4	.O8_h	2.6500	H13A	.O4_a	2.3300
H4	.C12_h	2.7800	H13A	.O6_a	2.7600
H4	.C13_h	3.0500	H13A	.C7_j	3.0900
H4	.H13C_h	2.5700	H13B	.C11_c	2.9400
H5	.O1_m	2.7100	H13B	.C7_j	3.1000
H5	.O8_h	2.7600	H13C	.H4_h	2.5700
H6	.C9_n	2.9800			

Table S9 - Hydrogen Bonds (Angstrom, Deg)

for: qo007 P -1 R = 0.02

C9 -- H9A .. O4	0.9800	2.5900	3.510(3)	157.00	2_666
C9 -- H9C .. O2	0.9800	2.5800	3.312(3)	131.00	2_776
C11 -- H11A .. O6	0.9800	2.5500	3.470(3)	157.00	2_565
C13 -- H13A .. O4	0.9800	2.3300	3.265(3)	159.00	2_665

Translation of Symmetry Code to Equiv.Pos

a =[2665.00] = 1-x,1-y,-z

b =[1545.00] = x,-1+y,z

c =[1655.00] = 1+x,y,z

d =[2776.00] = 2-x,2-y,1-z

e =[2676.00] = 1-x,2-y,1-z

f =[2666.00] = 1-x,1-y,1-z

g =[2565.00] = -x,1-y,-z

h =[2675.00] = 1-x,2-y,-z

i =[2786.00] = 2-x,3-y,1-z

j =[2775.00] = 2-x,2-y,-z

k =[1445.00] = -1+x,-1+y,z

l =[1455.00] = -1+x,y,z

m =[1565.00] = x,1+y,z

n =[1665.00] = 1+x,1+y,z

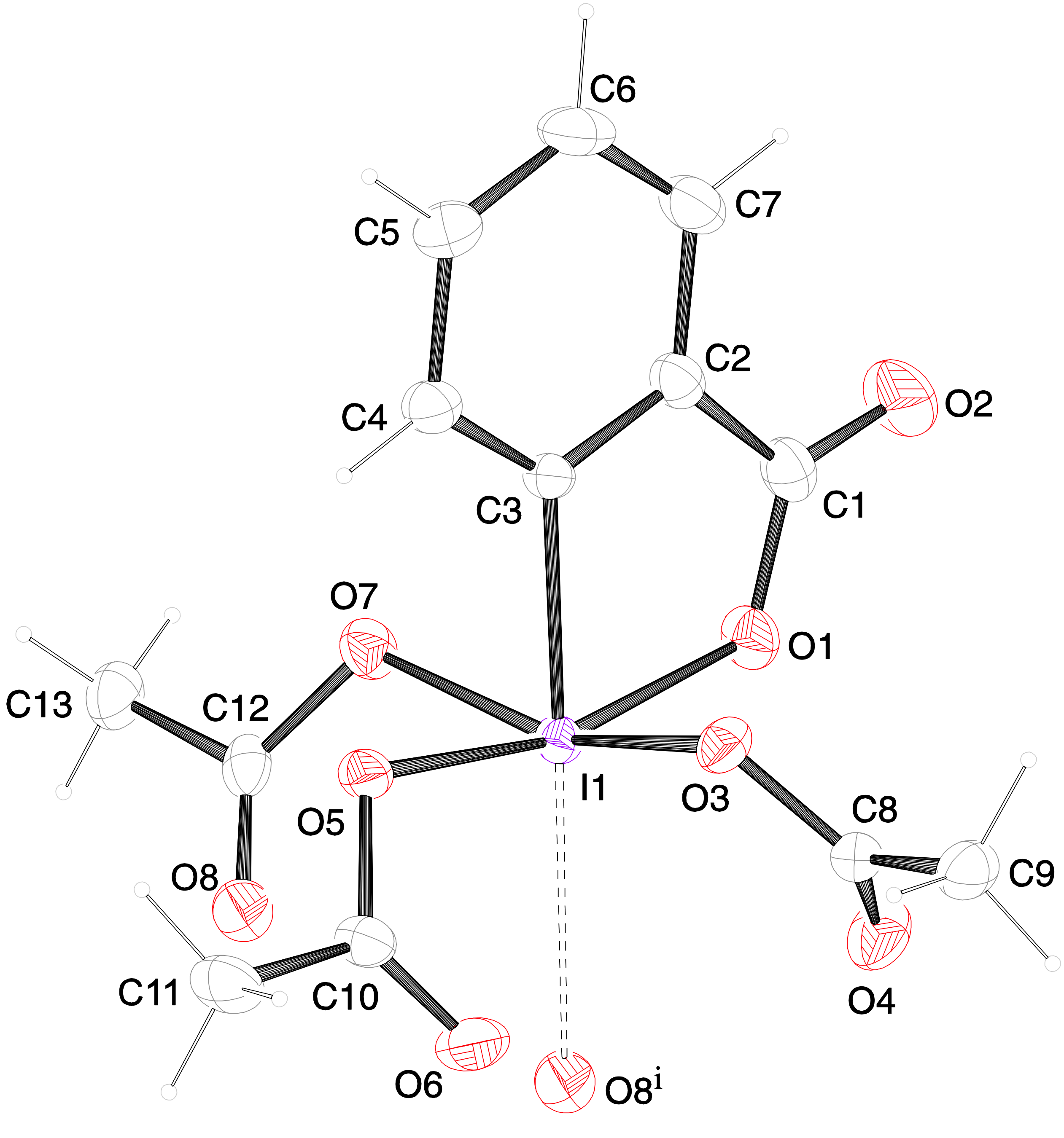
C6
C7
C5
C2
O2
C4
C1
C3
O7
O1
C13
C12
I1
O3
O5
C8
O8
C9
C11
C10
O4
O6
O8i
